# Active control of bright electron beams with RF optics for femtosecond microscopy

**DOI:** 10.1063/1.4999456

**Published:** 2017-08-21

**Authors:** J. Williams, F. Zhou, T. Sun, Z. Tao, K. Chang, K. Makino, M. Berz, P. M. Duxbury, C.-Y. Ruan

**Affiliations:** Department of Physics and Astronomy, East Lansing, Michigan 48824, USA

## Abstract

A frontier challenge in implementing femtosecond electron microscopy is to gain precise optical control of intense beams to mitigate collective space charge effects for significantly improving the throughput. Here, we explore the flexible uses of an RF cavity as a longitudinal lens in a high-intensity beam column for condensing the electron beams both temporally and spectrally, relevant to the design of ultrafast electron microscopy. Through the introduction of a novel atomic grating approach for characterization of electron bunch phase space and control optics, we elucidate the principles for predicting and controlling the phase space dynamics to reach optimal compressions at various electron densities and generating conditions. We provide strategies to identify high-brightness modes, achieving ∼100 fs and ∼1 eV resolutions with 10^6^ electrons per bunch, and establish the scaling of performance for different bunch charges. These results benchmark the sensitivity and resolution from the fundamental beam brightness perspective and also validate the adaptive optics concept to enable delicate control of the density-dependent phase space structures to optimize the performance, including delivering ultrashort, monochromatic, high-dose, or coherent electron bunches.

## I. INTRODUCTION

Electrons possess the highest scattering cross-section^1^ that could enable the development of high-performance beamlines,^2^ including the delivery of ultrashort electron bunches for broad ranges of ultrafast science investigation at a very high throughput. However, without the abilities to actively handle the space-charge-dominated beams, the unmitigated lengthening of bunches' time and energy spreads^3–5^ has limited the ultrafast electron diffraction (UED) system designs to seek optimization in the moderate-density and/or high-energy (relativistic) regimes,^6–9^ or in the realization of compact diffractometers.^10–12^ In the last decade, we have witnessed significant progress made regarding various UED techniques, for the study of gas phase molecules,^3,13^ surfaces,^14–16^ nanostructures,^17^ and nanometer thin films.^18–22^ The recent advances in the femtosecond (fs) regime^6,10,23^ from these continuous UED developments^24^ and the introduction of ultrafast electron microscope (UEM)^25^ are offering unprecedented capabilities for studying the atomic scale dynamics. Moreover, not restricted to the structural dynamics, the ultrafast spectroscopy^26–29^ through the UEM design offered new capabilities for obtaining spatially resolved electron dynamics. It is worth noting that to reach a sub-1 picosecond (ps) and sub-1 eV combined resolution, the current UEM systems employ only one or few electrons per bunch, which is complemented by a high repetition rate (∼MHz),^23^ nearly completely avoiding the onset of space-charge effects. Meanwhile, high-peak-intensity fs UED systems have recently been accomplished by incorporating the longitudinal optics, such as radio-frequency (RF) cavities,^30–32^ for electron bunch compression. This latest development is especially attractive for the future UEM design where the high-intensity implementation with longitudinal optics would be crucial to enable research on the irreversible processes,^33^ long-lived metastable phases,^34^ and for elucidating the transient bonding through ultrafast core-level spectroscopy.^35^ In particular, the chemically sensitive core-level regime suffers from the power-law suppression of the scattering cross-section,^36^ therefore, it is crucial to increase the bunch charge by several orders of magnitude from current UEMs to render it operable in probing long-lived states.

**FIG. 1.**
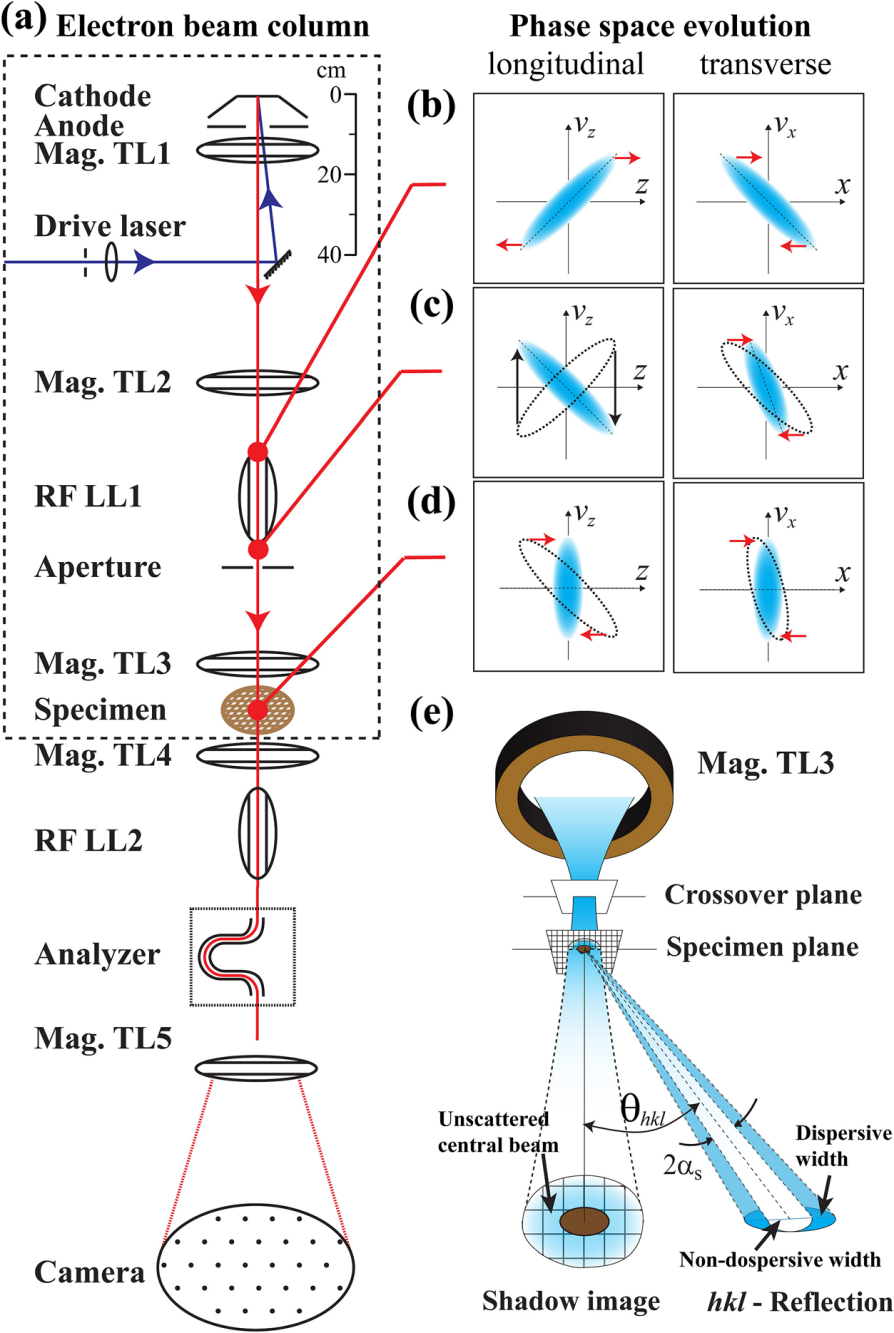
Ultrafast electron beam column with optics for controlling electron bunch phase space evolution. (a) Schematic drawing of a prototype UEM beamline. The focusing of the electron bunch generated from the photoelectron gun is handled by 5 magnetic transverse lenses (Mag. TL) and 2 radio frequency longitudinal lenses (RF LL). Ultrafast spectroscopy^38^ is accomplished by an energy analyzer (Analyzer) following the two RF lenses for time and energy compression. (b)–(d) The phase space evolution in the injector portion of the beamline (enclosed in dashed line). The focusing along the longitudinal (*z*) direction (left column) is controlled by RF LL1, adjusting the phase space structure in *(z, v_z_)*. Similarly, the transverse (*x,y*) focusing is controlled by Mag. TL2 and Mag. TL3 to manipulate the transverse phase space, e.g., in *(x, v_x_)*. In the phase space plots, the red arrows indicate the bunch's expansion or compression led by its internal velocity dispersion. The black arrows indicate the velocity shift driven by the RF field within LL1. (e) The conceptual outline of the atomic grating approach to characterize the energy spread of the electron bunches—for details, see the discussion in Sec. V.

Currently, an impediment that has prevented rapid advances in designing high-intensity UEM systems is the paucity of experimental data available on efficiently handling the space-charge-dominated beams. This is despite the recent success in implementing time-compression UED systems, where the main focus is to accomplish velocity bunching^37^ through an RF cavity. In a full-fledged UEM system that features diffraction, imaging, and spectroscopy in a single setup, the combination of different lenses is required to form ultrashort, monochromatic, high-dose, or coherent beams for different optimizations. Because of the relatively less known characteristics of the space-charge-dominated beams under different optical manipulations, their performance for the desired modalities is also unpredictable. This paper aims to address this issue through a combined experimental and theoretical approach to elucidate the key characteristics of space-charge-dominated beams. Our approach is based on the phase space perspective to elucidate the fundamental principle and limitations in focusing the high-intensity beams, including using a new optical design for manipulating the phase space of the electron bunches. The conceptual framework of this approach, especially the adaptive optical design involving the longitudinal lens pair for ultrafast electron spectroscopy [see Fig. 1(a)], has been discussed in detail previously.^38^ The work presented here focuses on the laboratory characterization of the bunch's phase space and emittance in the context of electron optics,^39^ which is the foundation for implementing the high-intensity UEM systems.

The advances discussed here include: the deployment of the atomic grating approach and theoretical models for characterizing phase space structures of the electron bunches; an understanding of the density-dependent bunch phase space structure evolution; control of bunch phase space through the RF cavity as a longitudinal lens; identification of high-brightness modes; and elucidation of the nonlinear effects induced at beam crossovers. It is worth mentioning that the ability of the RF lens to condense the beam's energy spread without significantly degrading the number of electrons delivered to the specimen provides an efficient new monochromatization scheme^36^ that may enable single-shot core-level ultrafast spectroscopy.^38^

This paper is structured into eight sections. Following this introduction, Sec. II discusses the realization of a high-quality factor RF lens and its control by a low-noise phase-locked loop at both low and high RF power levels for long-term stable operation. Section III surveys the current understanding of the multi-electron short-pulse emittance and brightness that fundamentally define the performance in the high-density beam regimes. In Sec. IV, the atomic grating concept to characterize the phase space structure and the RF optics is described. Section V discusses the practical strategies in realizing the phase space measurements using the atomic grating. In Sec. VI, comparative studies of the phase space structures for beams generated at different cathodes and at densities below and above the virtual cathode limit are presented. Section VII discusses the case study of photoinduced phase transition of nanoscale VO_2_ crystals with high-brightness beams. Section VIII summarizes the results and provides perspectives in improving the performance.

## II. DESIGN OF ELECTRON BEAMLINE AND RF LONGITUDINAL LENSES

The design of our electron beamline consists of a photoelectron gun and a series of electron optics to form a prototype UEM system as schematically illustrated in Fig. 1(a). The RF cavity is deployed as the longitudinal lens (LL) to actively control the beam longitudinal (*z*) phase space evolution. Meanwhile, the principle for transverse focusing using the transverse lens (TL) is similar to that already in place in conventional transmission electron microscopes (TEM), which utilize magnetic lens pair to adjust the transverse (*x* and *y*) phase space structure to form spatially coherent or focused probes.^39^ Our photoelectron gun employs the Pierce gun geometry where photoemission is driven on a flat silver photocathode over-coated onto a sapphire window by ultraviolet laser pulses (50 fs, 266 nm) through front illumination with a root-mean-square (RMS) radius of ∼90*μ*m.^40^ The average acceleration field between the cathode and the anode is 5 MV/m for delivering 100 keV electron bunches. For a given drive laser illuminated area, the number of emitted electrons in a single bunch, *N_e_*, depends on the drive laser power. Given that *N_e_* and the initial phase space structure are control parameters in our optical design, different photocathode coatings and laser powers are varied to explore the emittance and the brightness of the beams both below and above the virtual cathode limit,^41,42^ and compare their performance.

**FIG. 2.**
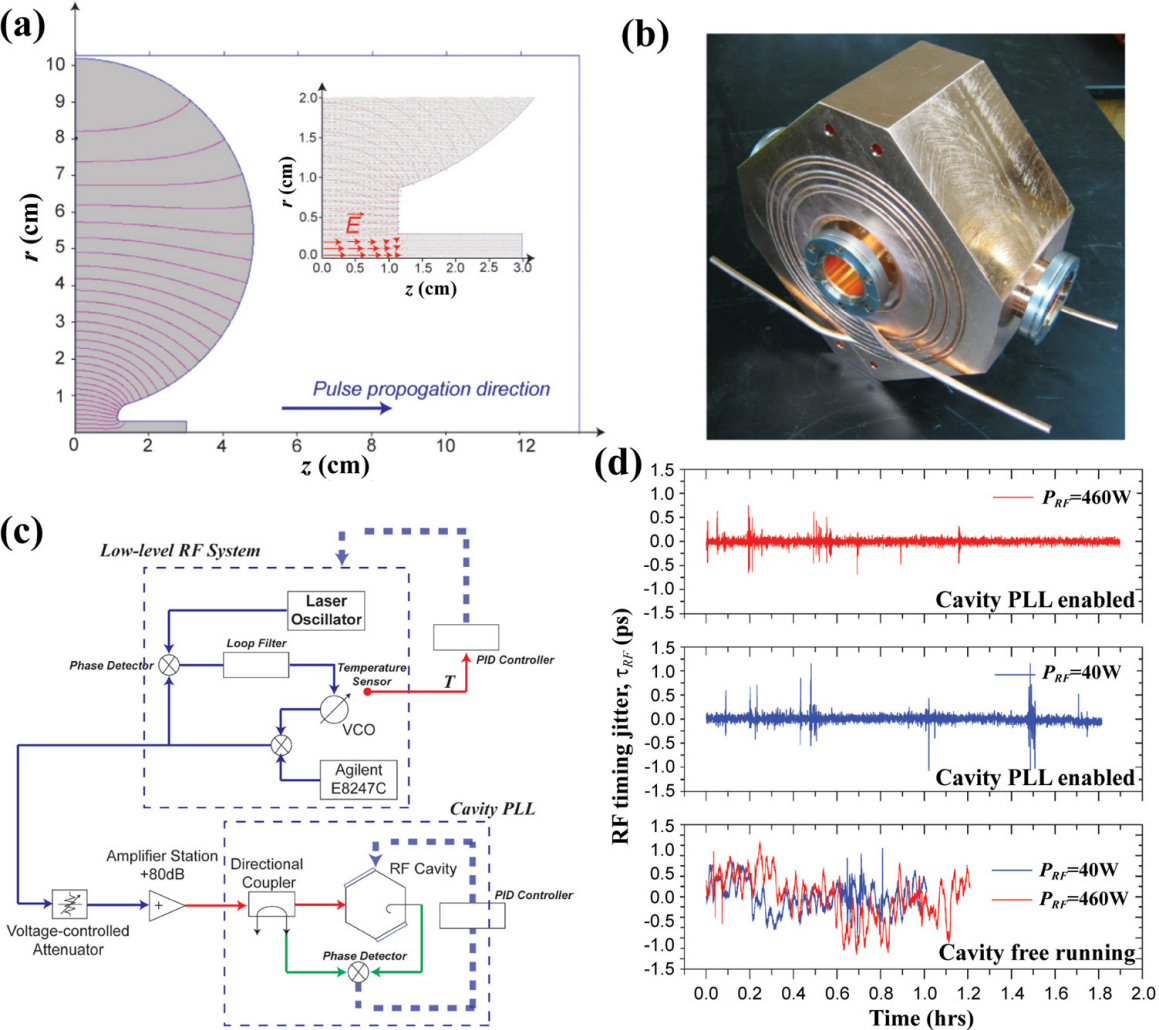
Design of RF cavity as a longitudinal lens. (a) Simulation result of the RF cavity field distribution with the Superfish code,^43^ plotted in cylindrical coordinates. Shaded area is the cavity chamber. Blue solid line is the equipotential lines, whereas the pink lines depict the electric-field distribution of the TM010 mode within the cavity. Inset: Enlarged view of the electric fields on the axis, along which the electron bunches propagate. (b) Picture of the copper RF cavity. (c) Schematic diagram for the overall RF control electronics setup, including the low-level and cavity phase-locked loops (PLL). The low-level PLL system ensures that the output signal is locked to the reference signal from the laser oscillator. The cavity PLL ensures that the RF cavity resonance frequency remains in-lock to the input signal from the low-level RF system by adjusting the cavity temperature through a PID controller. (d) The measurements of phase jitters at 460 W (top panel) and 40 W (middle panel) with the cavity PLL being activated. The lower panel shows the corresponding phase jitters without implementing the cavity PLL.

The properties of the high-brightness beam are characterized in the beam delivery system of the column [the region enclosed by the dashed line in Fig. 1(a)]. The RF cavity focuses the beam by adjusting the structure of the longitudinal phase space (*v_z_* vs. *z*) into a negative slope—see the left column along panels in Figs. 1(b)–1(d). Given that the leading edge of the electron bunch now has a more negative velocity than that of the tailing edge, the bunch will reach a compression in bunch duration at the specimen. In principle, this is the same as the magnetic focusing in terms of adjusting the transverse phase space (*v_x_* vs. *x*) [see the right column along panels in Figs. 1(b)–1(d)]. However, given the time-dependent nature of the RF field, it must be stable and be precisely timed relative to the arrival of the electron bunches at the cavity. Figure 2(a) shows the design of the RF cavity with its shape and electric field distribution calculated using the Superfish code.^43^ Figure 2(b) shows the picture of the copper cavity, after its machining is finished. The resonant frequency of the RF field *f_0_* is measured to be ∼1013 MHz and the quality factor (*Q*-factor) is ∼20k. The electric field of the TM010 mode is contained within the central region of the cavity^44^ [inset of Fig. 2(a)] and executes the compression. The average compressing field, *E_0_*, is defined as the integral of the *z* component of the field along the pulse longitudinal direction [Fig. 2(a)]. *E_0_* is related to the RF power, *P_RF_*, stored in the cavity. *E_0_* can be determined by measuring the energy change of the high-energy electrons, after they pass through the cavity, see the discussion in Sec. IV. The RF power can also be directly measured through the pick-up coil inside the cavity.

The electronic control of the RF cavity is separated into two independent systems: the low-level RF system and the cavity phase-locked loop (PLL), see Fig. 2(c). In the low-level RF system, the combined output of the voltage-controlled oscillator (VCO) and the RF synthesizer (Agilent E8247C) is synchronized with the laser oscillator pulse train at its 12th harmonics,^45^ serving as the reference frequency, *f_Ref_*, through a PLL. Additional timing drift is corrected with a feedback temperature control by stabilizing the temperature of the RF components. The output power of the low-level RF system is ∼ –11 dBm, which is then sent into the solid-state amplifier station with +80 dB gain. The power of the signal is adjusted by the voltage-variable attenuator before the amplifier station. At the cavity, the timing jitter due to the fluctuations of *f_0_* and *f_Ref_* is further corrected by the cavity PLL, which actively tunes the cavity temperature and forces *f_0_* to follow the changes in *f_Ref_*. To control the cavity resonance, four thermoelectric modules are mounted on the cavity serving as temperature actuators. The voltage signal from the phase detector is fed back to a PID controller (Thorlab TED4015), which drives the thermoelectric modules to actively adjust *f_0_* by changing the cavity temperature. The stabilities of the timing and the power at the RF cavity are controlled at ≤50 fs and ≤10^−3^ levels with active sub-50 mK temperature stabilization in the electronics and at the cavity. By doing so, *f_0_* can actively track the change in the input frequency from the low-level PLL and keep the cavity always on resonance. Figure 2(d) shows the importance of the cavity PLL in stabilizing the RF timing jitter, *τ_RF_*, especially when the temperature of the room (to be renovated) can vary up to 1 °C over a long-term operation. The RF timing jitter leads to the relative shift of the cavity electric field phase to the arrival of the electron pulses, *Δϕ*. This effect can cause energy gain or loss in the electron pulse, and hence an arriving time jitter, *τ_Ar_*, of the electron pulses. When the cavity PLL is turned on, the low-frequency fluctuation is completely suppressed, which is an obvious improvement compared to the free-running cavity.

## III. DENSITY-DEPENDENT PHASE SPACE STRUCTURE EVOLUTIONS: THEORETICAL PREDICTIONS

In a UEM equipped with active optics for phase space manipulation, the crucial manifestation of space charge effects is of stochastic nature and provides fundamental limits to the achievable space, time, and energy resolutions. This stochastic space charge effect is caused by an irreversible growth in RMS beam emittance^46^ due to the fluctuating components of the nonlinear electron dynamics. In the UEM, it is correlated with virtual cathode formation during the electron pulse generation. The virtual cathode limit (VCL)^41,42^ sets in due to the attractive image charge field on the photocathode surface left by the emitted electrons, resulting in an increased fraction of recombined electrons and a degradation of beam properties once the critical current is reached.^47^ Below the VCL, the high-density electron bunches created at the photocathode also develop unique phase space structures depending on the initial conditions.^42,48^ Highly relevant to the current studies is the prediction based on the multi-level fast multiple method (MLFMM)^49^ simulations^42,50^ that the stochastic scattering at the initial stage leads to a sublinear growth of the transverse phase space area, or emittance (*ε_x_* and *ε_y_*), with respect to *N_e_*, whereas the terminal longitudinal emittance (*ε_z_*) is nearly linear,^42^ until the VCL is reached. This prediction implies that it is favorable to extract the high-intensity beams perceived in high-throughput UEM systems close to the VCL to render beams with a high transverse brightness, defined by *N_e_*/emittance, while maintaining reasonable longitudinal emittance to reach desired temporal and spectral resolutions.^38^

For self-consistency purpose between experimental measurements and theoretical predictions, the normalized emittance in the *z* direction *ε_z_* is defined as^46^

$$\varepsilon_{z}=\frac{1}{m_{e}c}\sqrt{〈z^{2}〉〈p_{z}^{2}〉-{〈zp_{z}〉}^{2}},$$ (1)

where the brackets ⟨⋯⟩ stand for an average over the ensemble of electrons in the bunch over coordinates of position (*z*) and momentum (*p_z_*). Equation (1) can be reduced to the expression

$$\varepsilon_{z}=\frac{\gamma \sigma_{z}\sigma_{vz}}{c}$$ (2)

at the beam waist, where *σ_z_* is the RMS bunch length, *σ_vz_* is the RMS of velocity distribution, and *γ* is the relativistic Lorentz factor. The normalized emittance can thus be understood as the area the electron bunch occupies in its phase space. In this case, *σ_z_* and *σ_vz_* are the projections of the phase space along the *z* and *v_z_* axes. Similarly, the normalized emittance in the *x* and *y* directions can be defined by replacing *z* with *x* or *y* in Eqs. (1) and (2). According to Liouville's theorem, the emittance should be conserved throughout the beam propagation. In other words, the ultimate beam brightness deliverable on the sample plane is limited by the terminal electron-beam emittance after fully extracting the beam from the cathode in an ideal situation.

**FIG. 3.**
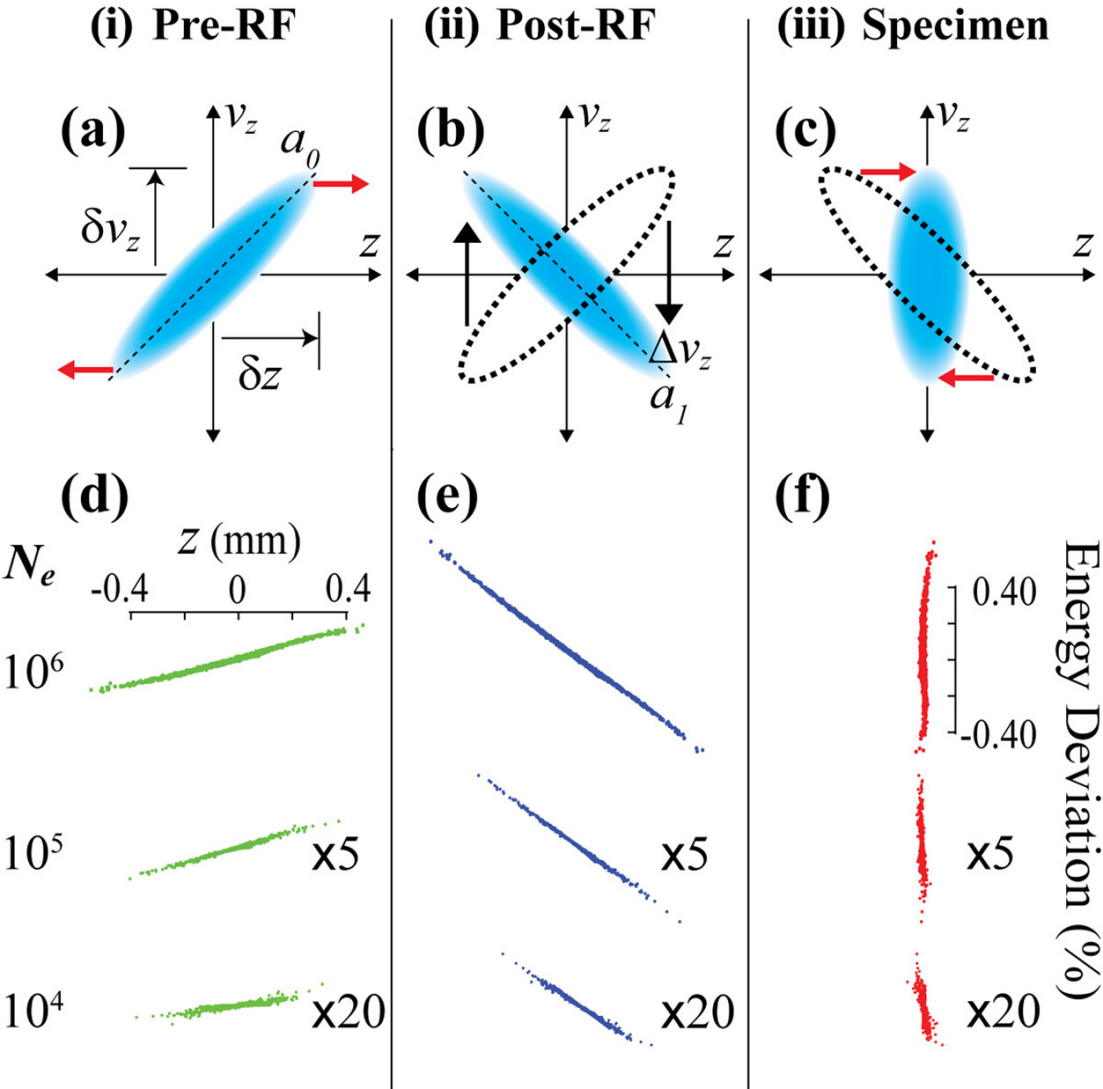
Longitudinal phase space evolution of the electron bunch under RF lens focusing. (a)–(c) Depiction of the evolution of the electron bunch longitudinal phase space in a 2D Gaussian: (i) right before and (ii) right after the RF cavity, and (iii) at the sample plane to achieve time. The chirp $a\equiv \delta v_{z}/\delta z$ is defined as the slope of the phase space, taken using the projected components $\delta v_{z}$ and δz. The red arrows indicate the bunch's expansion or compression led by its internal velocity dispersion. The black arrows indicate the velocity adjustment $\Delta v_{z}$ made by the RF field, shifting the chirp from *a_0_* to *a_1_*. (d)–(f) MLFMM beam dynamics simulations for *N_e_* = 10^6^, 10^5^ and 10^4^ showing the dependence of the bunch length and chirp evolution at the three stages outlined by panels (a)–(c) over different *N_e_*. Increased nonlinearity at stage (iii) can be seen for higher *N_e_*. A closer look is shown for *N_e_* = 10^6^ in Fig. 9(b). For a better comparison to the case of *N_e_* = 10^6^, the phase space structures associated with *N_e_* = 10^5^ and *N_e_* = 10^4^ are scaled up by factors 5 and 20, respectively, on both transverse and longitudinal scales.

To facilitate the analytical formulation, the bunch phase space is described in an idealized two-dimensional (2D) Gaussian in terms of position (*z*) and velocity (*v_z_*), as shown in Fig. 3(a). In this representation, the phase space chirp, *a_z_* ≡ *dv_z_/dz*, is defined as the slope of the principle axis of the 2D structure.^51,52^ To more easily compare with the measurements, here, the phase space is described in the relative laboratory frame instead of the center-of-mass (CoM) frame,^38^ namely, *v_z_* and *z* are the coordinates after subtracting the CoM velocity and position. Figures 3(a)–3(c) depict the snapshots of the phase space evolution during RF compression. Just before the RF cavity [pre-RF stage (i)], the phase space has a positive chirp, representing an expanding electron bunch. The role of the RF field in longitudinal focusing is to exert a proportional velocity shift *Δv_z_* to the particle in the bunch that shifts the chirp from a positive *a_0_* (pre-RF) to a negative *a_1_*, as represented in panel b just after the cavity [post-RF) (ii)]. The resulting negative velocity gradient leads to the compression of the electron bunch during flight to the specimen (panel c). The MLFMM simulations provide the corresponding N-particle depiction of the phase space structures. The simulations are performed for different *N_e_*'s at a fixed beam kinetic energy, *K_0_*, of 100 keV, as shown in Figs. 3(d)–3(f). Specifically, the RF compression is performed using the RF module in COSY INFINITY,^53^ where the voltage of the cavity is described by a sinusoidal function with position-dependent energy gain (or loss) occurring over an infinitesimally thin region. Macroparticles are used to solve for large numbers of electrons (*N_e_*≥10^5^) after checking that the N-particle simulations remain consistent using this approximation. The dynamical equations were solved with 4th order Runge-Kutta integrators.^50^ For different *N_e_*'s, the phase space develops into different sizes and chirps. Nonetheless, the phase space largely maintains a linear structure during the compression. The performance, as measured by the RMS bunch spreads in *z* and *v_z_*, is limited by their respective emittance. A linear correlation between the measured RMS time (*Δt*) and energy (*ΔE*) spreads and the phase space projections can be made: *Δt *=* k_t_σ_z_*, *ΔE *=* k_v_σ_vz_* where at 100 keV, *k_t_* and *k_v_* are 0.00608 ps/*μ*m and 1597 eV/(*μ*m/ps), for converting into resolutions in the laboratory frame. The time and energy spreads of ∼50 fs and ∼400 eV at the focal plane (specimen) are accomplished for *N_e_* = 10^6^, whereas for a lower *N_e_*, the performance is significantly improved, e.g., sub-10 fs and sub-10 eV, with *N_e_* = 10^4^ at the temporal focal plane.

## IV. ATOMIC GRATING APPROACH FOR PHASE SPACE CHARACTERIZATION

Here, we describe the experimental strategies to characterize the electron bunch's phase space and the theoretical framework linking the measured phase space parameters to the optical manipulation required for focusing in time and energy. Our approach is based on the atomic grating, namely, utilizing the atomic crystals to sort out the incident beam's energy and, after optical activation, time spreads, in order to reconstruct the phase space. For reasons that will become apparent later, we center our formulation of the atomic grating approach on a key RF focusing parameter *η*, defined by the beam velocity change (δ*v_e_*) observed under an RF phase tuning (*δϕ*):

$$\eta \equiv -\frac{1}{v_{e}}(\frac{\delta v_{e}}{\delta \phi }).$$ (3)

Given that *η* is proportional to the RF field strength used to shift the velocity, resulting in the chirp change *Δa* (see Fig. 3), it can be established that

$$\Delta a=-\frac{2\pi \;f_{0}\;\eta }{\;\text{cos}\;\phi },$$ (4)

where *f_0_* is the RF cavity resonance frequency. Equation (4) can be derived from Eq. (3) by considering that the RF-induced velocity shift δ*v_e_* is proportional to the relative phase of the particle to the CoM of the bunch, namely, $\delta \phi =\left(\delta z/v_{e}\right)\cdot 2\pi f_{0}$. Furthermore, introducing the time-dependent RF field $E\left(t\right)=E_{0}\;\text{sin}\;\left(2\pi f_{0}t+\phi \right)$, the relationship between *η* and the RF parameters can be formulated:

$$\eta =\frac{eE_{0}}{\gamma^{3}\pi \;f_{0}v_{e}m_{e}}\text{sin}\left(\frac{\pi \;f_{0}\;d}{v_{e}}\right)\text{cos}\;\phi ,$$ (5)

where *e* and *m_e_* are the electron charge and the rest mass, and *d* is the RF gap length. See Table I for the typical values of parameters employed in our setup.

**TABLE I.** Notations and key operating parameters.

Parameters	Symbols	Typical values (unit)
Number of electrons in a single electron bunch	*N_e_*	10^6^–1.7 × 10^7^
Normalized emittance in *x,y,z*	*ε_x,y,z_*	0.002–2 (*μ*m) or (mm·mrad)
Beam kinetic energy	*K_0_*	100 (keV)
Beam velocity	*v_e_*	164.35 (*μ*m/ps)
Beam energy spread, RMS	*ΔE*	10–1000 (eV)
Beam time spread, RMS	*Δt*	0.1–30 (ps)
Beam divergence angle, HWHM	*α*	(degree) or (rad)
Scattering angle for *hkl* reflection	*θ_hkl_*	(degree) or (rad)
Relativistic Lorentz factor	*γ*	1.1957
Electron de Broglie wavelength	*λ_e_*	0.0037 (nm)
RF cavity phase	*ϕ*	(degree)
Applied RF power	*P_RF_*	0–400 (W)
RF focusing parameter	*η*	0–0.003 (degree^−1^)
RF longitudinal lens coefficient	*k_LL_*	5.65 × 10^−4^ (ps^−1^ W^−1/2^)
RF cavity electric field strength at gap	*Ε_0_*	0–1.5 (MV/m)
RF gap length	*d*	2.1 (cm)
RF cavity resonance frequency	*f_0_*	∼1.013 (GHz)
RF phase locked loop reference frequency	*f_Ref_*	1.01254 (GHz)
RF cavity to specimen distance	*L*	0.425 (m)
Specimen to camera distance	*L_Cam_*	0.370 (m)
RF timing jitter, RMS	*τ_RF_*	∼45 (fs)
Electron bunch arrival time jitter, RMS	*τ_Ar_*	≤100 (fs)
Coherence length	*L_C_*	10–30 (nm)
Grating dispersion power	*k_D_*	0.103 (*μ*m/eV) @ *s* = 10 Å^−1^

**FIG. 4.**
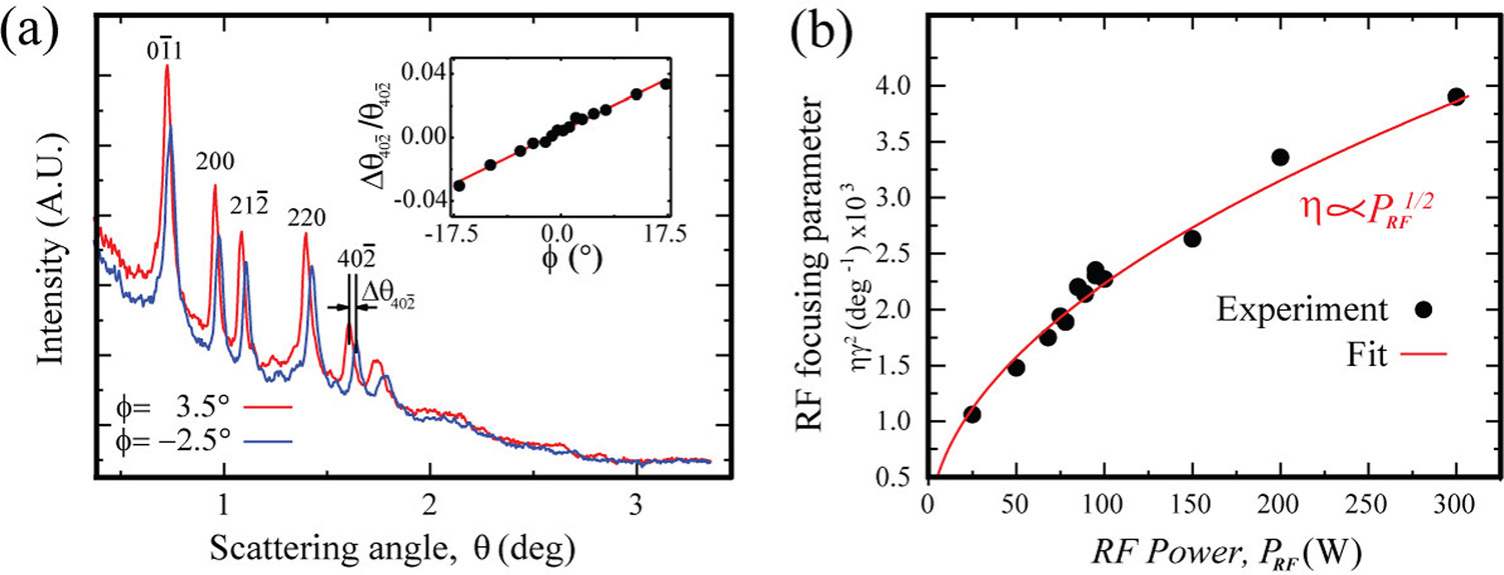
Characterization of the RF focusing parameter *η*. (a) The angular shift of the diffraction curves recorded on the camera induced by an adjustment of the RF phase (*ϕ*) taken at an RF power *P_RF_* = 75 W. The specific angular shift ($\Delta \theta_{40\bar{2}}$, as compared to the angle at *ϕ* = 0°) associated with the $40\bar{2}$ reflection is utilized to determine the focusing parameter *η*. As shown in the inset, the normalized angular shift $\Delta \theta_{40\bar{2}}/\theta_{40\bar{2}}$ is proportional to the RF phase *ϕ. Τ*he slope of the curve is equal to *ηγ^2^*, where γ is the relativistic Lorentz factor. (b) The measured *ηγ^2^* versus the applied RF power *P_RF_* (solid symbols). The curve fit (red line) is based on the equation: $\eta \gamma^{2}=kP_{RF}^{1/2}$, where the coefficient *k* is used to determine the RF lens coefficient (see the discussion in Sec. IV).

We demonstrate the ability to determine *Δa* and *E_0_* through the measurement of *η* using the VO_2_ thin film as the atomic grating. Figure 4(a) shows the results obtained by passing the electron bunch through the grating in two different RF phases: *ϕ* = −2.5° and 3.5°. In each case, the diffraction curve is obtained from a line scan along the center of the diffraction image acquired by the pixilated CCD camera, where each pixel is converted into the scattering angle *θ*. The clearly visible angular change, *Δθ_hkl_*, from the *hkl* reflection throughout the curve is the result of bunch velocity change induced by adjusting the RF phase. This can be formulated in terms of normalized changes: $\Delta \theta_{hkl}/\theta_{hkl}=-\gamma^{2}\Delta v_{e}/v_{e}$, where *θ_hkl_* is the scattering angle of *hkl* reflection. This formulation is valid strictly only in the small angle regime, where given the scattering wavevector associated with a specific *hkl* reflection $s_{hkl}=4\pi /\lambda_{e}\cdot \;\text{sin}\;\left(\theta_{hkl}/2\right)$ is unchanged, the normalized change in *θ_hkl_* is proportional to that of the de Broglie wavelength, $\lambda_{e}=h/(\gamma m_{e}v_{e})$, where *h* is Planck's constant, or in other words, the normalized change of *v_e_*. Specifically, the inset of Fig. 4(a) shows the linear correlation between $\Delta \theta_{40\bar{2}}/\theta_{40\bar{2}}$ and *ϕ*, where the slope of the curve is equal to *ηγ*^2^.

To demonstrate that the RF cavity can be employed as a longitudinal lens, a robust relationship between the applied cavity power *P_RF_* and the resulting phase space adjustment *Δa* must be demonstrated. An empirical correlation between *P_RF_* and *Δa* is established by measuring *η* as a function of *P_RF_* with the aforementioned approach. Our results, as reported in Fig. 4(b), show a square-root dependence between the two. By fitting the data with the equation: $\gamma^{2}\eta =kP_{RF}^{1/2}$, we can determine the coefficient *k*, which is then translated, via Eq. (4), to establish the relationship between *Δa* and $P_{RF}^{1/2}$ – specifically $\Delta a=k_{LL1}\cdot P_{PF}^{1/2}$, where $k_{LL1}=5.65\times 10^{-4}$ ps^−1 ^W^−1/2^ is defined as the RF lens coefficient.

In the following, we show that in the non-interacting scenario, namely, when the space-charge effect does not further modify the phase space structure during compression, a simple geometric relationship can be established between time and energy compressions. First, for reaching the time compression point *η_t_*, the chirp is shifted from a specific *a_0_* (pre-RF) to $a_{1}=-v_{e}/L$ (post-RF), or

$$\eta_{t}=\frac{\left(a_{0}+\frac{v_{e}}{L}\right)}{2\pi \;f_{0}},$$ (6)

where *L* is the distance between the RF lens and the specimen. On the other hand, for reaching the energy compression point *η_E_*, the chirp is shifted to *a_1_* = 0, or

$$\eta_{E}=\frac{a_{0}}{2\pi \;f_{0}}.$$ (7)

Therefore, Δ≡*η_t_* –*η_t_* = *v_e_*/(*2πf_0_L*) is purely geometrical and independent of *P_RF_* or the incident beam parameters. One central aspect of our work is to verify if the simple linear RF focusing principles described by Eqs. (6) and (7) indeed apply to the incident beams at different densities and preparations. The assurance of the linear responses that satisfy both Eqs. (6) and (7) will permit us to project performances under various longitudinal optical settings, including forming a lens pair for conducting the spectroscopy.^38^ On the other hand, to understand the limits of compression, one needs to obtain information on the emittance. For evaluating the beam emittance, we can examine the projected parameters at the crossovers (compression points), where the product of the measured *σ_z_* and *σ_vz_* gives the normalized emittance as described by Eq. (2).

## V. EXPERIMENTAL STRATEGIES

We discuss further implementation of the atomic grating approach for determining the phase space parameters, through the measurements of the energy and time spreads of the bunch and the critical RF power required to achieving compressions. First, on obtaining the energy spread of the bunch, the atomic grating is employed as an energy analyzer, through which the energy spread translates into the lateral broadening of the diffracted beams, as described in Fig. 1(e). We look at the dispersive component of the diffraction width (dispersive width), *σ_E_*, to extract *ΔE*, which is related to the grating dispersion power, *k_D_*,^36^ namely, $\sigma_{E}=k_{D}\Delta E$. We note that for an atomic grating, *k_D_* is direction- and angle-dependent. Here, for simplicity, we restrict our analysis along the radial (*r*) direction, and express it in terms of scattering wavevector *s*:

$$k_{D}\equiv \frac{dr}{dE}=\left(\frac{\gamma }{1+\gamma }\right)\frac{2L_{Cam}\;\lambda_{e}\;s}{K_{0}{\left[4\pi^{2}-{\left(\lambda_{e}s\right)}^{2}\right]}^{1/2}},$$ (8)

where *L_Cam_* is the camera distance. Meanwhile, the non-dispersive component of the width (nondispersive width), *σ_ND_*, which is independent of *s*, is subject to the transverse optics settings and the quality of the grating to form sharp diffraction patterns.^38^ Combining the two components, we can express the observed full diffraction width, *σ_Β_*:

$$\sigma_{B}={\left(\sigma_{E}^{2}+\sigma_{ND}^{2}\right)}^{1/2}.$$ (9)

Therefore, from Eqs. (8) and (9), in principle, we can extract *ΔE* from an arbitrary diffraction peak at *s_hkl_*:

$$\Delta E=K_{0}\left(\frac{1+\gamma }{\gamma }\right)\frac{\sqrt{\sigma_{B}^{2}\left(s_{hkl}\right)-\sigma_{ND}^{2}}}{s_{hkl}}.$$ (10)

Nonetheless, in order to achieve the highest possible energy resolution, the contribution from the nondispersive components should be minimized. For example, the optical width can be reduced via a proper transverse optical arrangement, such as using a magnetic lens pair to produce a tight parallel beam illuminating a small specimen. Other contributions to *σ_Β_* include the point spread function (PSF)^38^ of the camera and the inherent diffraction width that originated from the finite persistence length of the crystalline lattice. The inherent diffraction width may be treated as a fitting parameter or essentially ignored by employing high-quality single-crystal samples as the grating. Because *k_D_* roughly scales with *s*, a large *s* is preferred in the analysis. By adjusting the transverse optics and choosing high-quality single crystals as the atomic grating, we could reach a resolving power at the level of 10^−3 ^Å^−1^ or an energy resolution at the level of 10 eV or below, depending on the quality of the data. This level of resolution is capable of discriminating 10 nm or less in normalized longitudinal emittance for identifying high-brightness beam generation.^38^

**FIG. 5.**
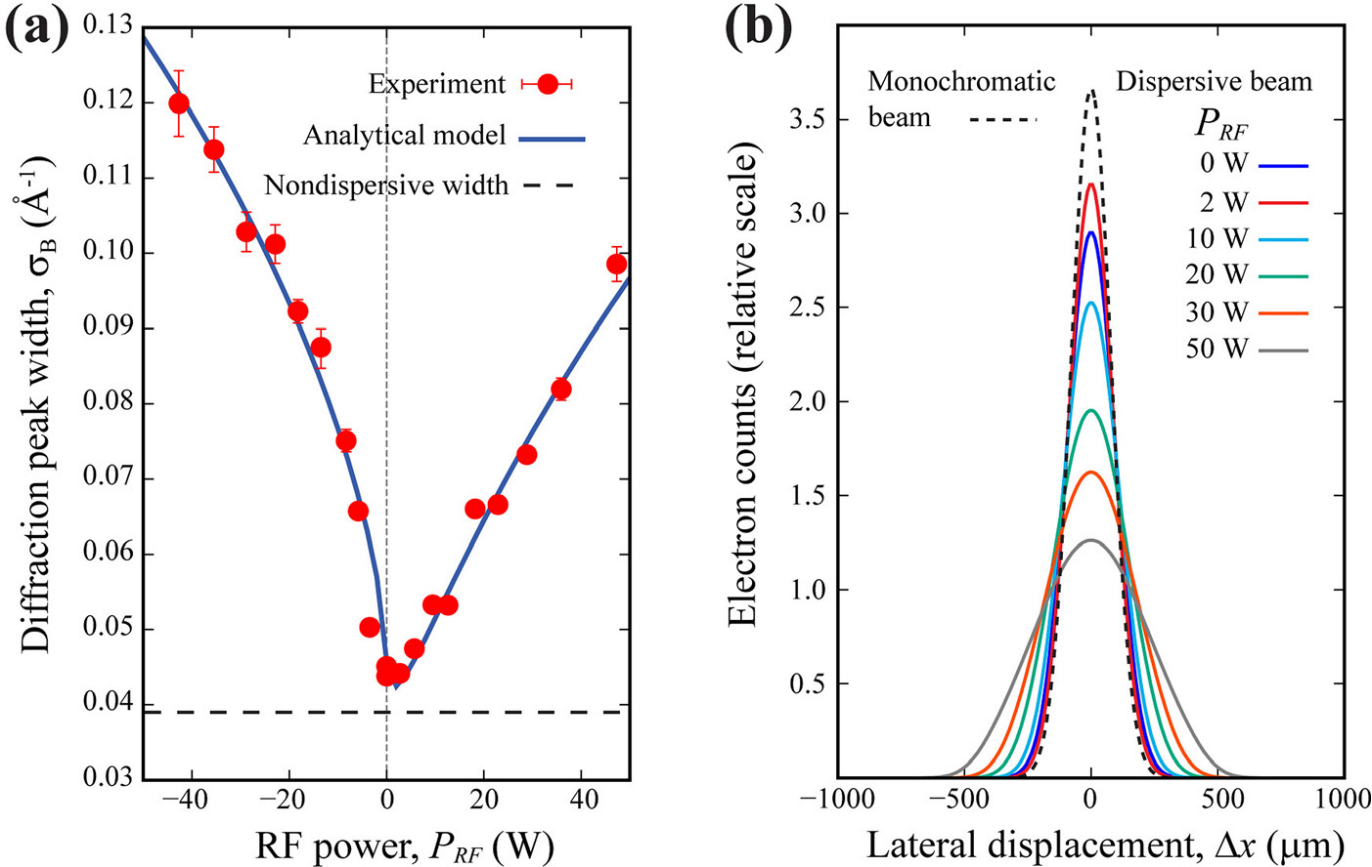
Measurements of diffraction width to determine the bunch energy spread under different RF cavity powers with the atomic grating approach. (a) The measured RMS diffraction width *σ_B_* at different RF powers (solid symbols) for *N_e_* = 1.7 × 10^7^. The “negative” power represents bunch stretching cases when the RF phase is set to 180°. The solid line shows the fitting of the experimental data using the analytical model, where the fitting parameters: *ε_z_*, *a_0_*, and *σ_0_* are listed in Table II. (b) The calculated diffraction profiles from the analytical model under different RF power settings. The horizontal axis is the lateral displacement from the center of the diffraction peak. The different widths are caused by different energy spreads when the chirp of the phase space is shifted by the RF field. The dashed line shows the profile calculated for the monochromatic beam with no energy spread. Its width represents the nondispersive width indicated in (a).

To demonstrate the principle of this technique, we conducted the measurements using the electron beams generated from an ultrathin silver photocathode coating (≤10 nm) and employed a free-standing single-crystal TaS_2_ film^21^ as the grating. We pushed *N_e_* beyond the VCL for demonstrating a large beam emittance. In this case, the employed *N_e_* is 1.7 × 10^7^ (determined by electron counting^40^), which is just above the critical number, *N_c_*, of 1.5 × 10^7^ determined at the VCL. We used TL2 to first create a crossover close to TL3, then used TL3 to form a parallel beam at the specimen. This results in an optical width^38^ of 0.039 Å^−1^, which is the major contributor for *σ_ND_*. Next, we recorded the RF power-dependent *σ_B_* as reported in Fig. 5(a). Specifically, to provide an adequate baseline for comparison, the RF phase was first set to out-of-phase (ϕ = 180°), which results in the increase of the chirp [see Eq. (4)], and then switched to in-phase (ϕ = 0°), which results in the decrease of the chirp. This strategy provides the power dependence on both sides of the curve shown in Fig. 5(a), where the “negative” *P_RF_* represents the out-of-phase condition, whereas the “positive” *P_RF_* represents the in-phase condition. The reported diffraction RMS widths (*σ_Β_*, square symbols) are obtained by (Gaussian) fitting the individual Bragg peak profile retrieved at *s *=* *8.7 Å^−1^. The experimental data are compared to the values extracted from the simulated profiles [Fig. 5(b)] produced using the analytical 2D phase space structure, where normalized emittance *ε_z_*, pre-RF chirp *a_0_*, and bunch width *σ_0_* are the fitting parameters. The simulation of the diffraction profile after the grating is produced by convoluting the phase space structure function with the analyzer transfer function using the approach described in Ref. 38. Here, we use the grating dispersion power *k_D_* = 0.103 *μ*m/eV calculated from Eq. (8), and the changes in the diffraction width are rather visible. The lowest width is obtained at ∼1 W, which corresponds to the energy compression point where the chirp is zero. Away from 1 W, the width is increased due to a nonzero chirp, which increases over the RF power or *η* [see Fig. 5 and Eq. (4)] under both compressing (positive) and stretching (negative) conditions. From fitting the data (solid line), we obtain *a_0_ =* 0.00010 ± 0.00002 ps^−1^, *ε_z_* = 1.5 ± 0.5 *μ*m, and *σ_0_* = 18 ± 2 ps. The projected time compression point [from Eq. (6)] in this case is near 50 W.

A complementary view of the phase space can be obtained by measuring the time spread of the bunch as a function of RF power. The measurements can be conducted within the atomic grating framework already implemented for the energy compression experiments where an additional laser pulse is deployed as a pump to drive the change in the grating structures. Hence, the bunch duration can be extracted from the pump-probe cross-correlation time, assuming that the inherent dynamics is shorter than the bunch duration. This is in essence the resolution-limited UED experiment implemented on the grating specimen. A key advantage of this approach is that the phase space structure as represented by its projection along the time and energy axes can be simultaneously determined. This is important to correlate the energy and time compression data to examine the linear response of the phase space over the RF optics, which will be discussed in detail in Sec. VI for different cases of photoemissions.

## VI. DENSITY AND SOURCE-DEPENDENT PHASE SPACE STRUCTURES AND PERFORMANCE

**FIG. 6.**
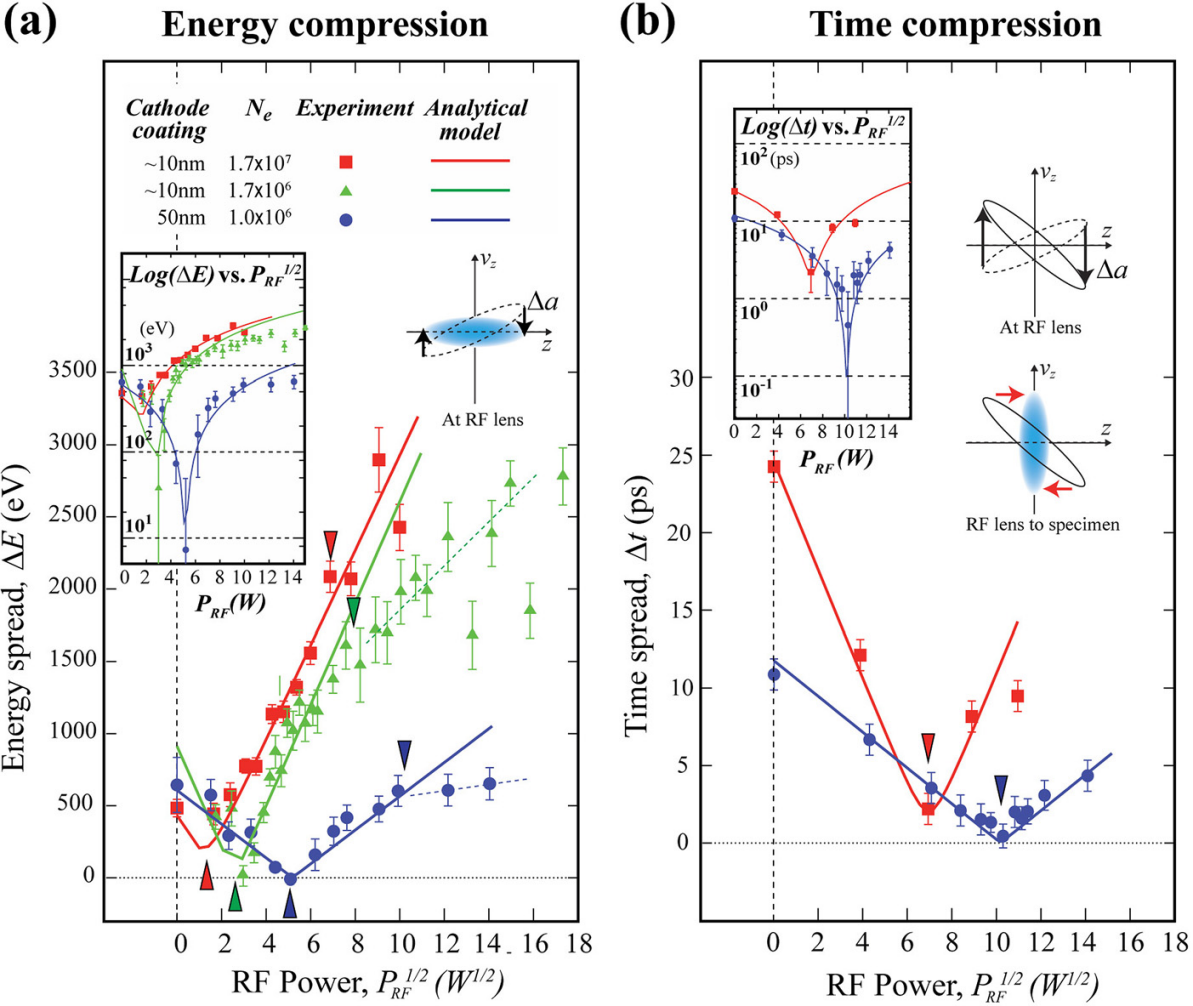
Energy and time compressions by the RF lens under different photoemission conditions. (a) The bunch RMS energy spread (*ΔE*) obtained at different RF powers presented on the scale $P_{RF}^{1/2}$. The solid symbols are the experimental results under different scenarios of photoemission, whereas the lines are fits to the experimental results using the analytical model. The compression points (marked by the upward triangles) are reached when the phase space tilt is along the *z* axis. The resulted chirp change (*Δa*), see the cartoon inset, is proportional to $P_{RF}^{1/2}$. (b) The bunch RMS time spread (*Δt*) presented on the scale $P_{RF}^{1/2}$. The temporal compression occurs when the final phase space tilt is along the *v_z_* axis – see the inset cartoon depiction of the phase space evolution. The red arrows indicate the self-compression of the bunch from the RF lens to the specimen after a negative chirp is established. For both panels, the downward triangle marked the locations of the time compression points, and the insets (on *log* scale) highlight the differences in achievable bunch compression.

Applying the aforementioned strategies, we surveyed the phase space structures in different regimes of photoemission to identify high-brightness modes, including employing different thicknesses of photocathode coating and density regimes that are both below and above VCL. These comparison studies were performed using the high-quality TaS_2_ thin film as the grating. First, as reported in Fig. 6(a), we examined the energy spread from bunches generated using the ultrathin photocathode coating (≤10 nm, also reported in Sec. V). We present the measurements both above (*N_e_* = 1.7 × 10^7^, solid squares) and below (*N_e_* = 1.7 × 10^6^, solid triangles) the VCL (*N_c_* = 1.5 × 10^7^). For the other photocathode coating (50 nm), the study was conducted for *N_e_* = 10^6^ (solid circles) which is below the VCL. The reported *ΔE* values are converted from the measured and simulated widths following the procedures in Sec. V. In addition, we also conducted the UED measurements to determine the corresponding *Δt* of the bunches at different RF powers, as reported in Fig. 6(b).

In the case of the ultrathin photocathode, the above-VCL emission has the lowest energy spread at ∼300 eV, while the below-VCL emission has the lowest energy spread well below 100 eV. Furthermore, the compression points for the two cases are also very different. The energy compression for the above-VCL case occurs near $P_{PF}^{1/2}∼1\;W^{1/2}$ (or *P_RF_* ∼ 1 W), whereas for the below-VCL case, it occurs at $P_{PF}^{1/2}∼2.3\;W^{1/2}$ (or *P_RF_* ∼ 5.3 W). The different energy compression points reflect different *a_0_*. In particular, the lower Δ*a* required for energy compression observed at above-VCL suggests its more rapid expansion in the bunch width than in the energy spread during the beam formation stage. Nevertheless, the generally higher energy spread observed at above-VCL also indicates a higher emittance than at below-VCL. The most non-intuitive observation is probably that a lower incident energy spread at *P_RF_* = 0 is obtained at above-VCL rather than at below-VCL, despite its higher emittance. This phenomenon can only be reconciled with the much lower pre-RF chirp developed for the bunches at above-VCL, which is not expected if only the magnitude of the collective space charge force is considered for driving the spreading of the bunches.

In contrast, a much better performance is accomplished using the 50 nm photocathode coating as indicated in the solid circles in Fig. 6(a). The relative energy spread (*ΔE/K_0_*) in this case (*N_e_* = 10^6^) is well below 1% throughout the range of measurements, which is significantly below the level observed using the ultrathin photocathode. The improvement is also seen in the lowest energy spread possible (≤10 eV, resolution-limited) accomplished at $P_{PF}^{1/2}∼5\;W^{1/2}$ or *P_RF_* ∼ 25 W, a much higher power than the previous cases. The best fits (solid lines) to the data using the analytical model show very different *ε_z_* and *a_0_* values (see Table II). The much higher emittances obtained for the ultrathin photocathode (*ε_z_* = 1.5 and 0.4 *μ*m, respectively) as compared to the thicker (50 nm) photocathode (*ε_z_* = 0.03 *μ*m) and their significantly different chirps strongly indicate the highly sensitive nature of the phase space evolution before reaching the steady state depending on the initial conditions. This sensitivity to the initial conditions is especially noticeable when comparing the beam emittances from the two photocathodes (0.4 *μ*m vs. 0.03 *μ*m) using a similar number of electrons (1.7 × 10^6^ and 1.0 × 10^6^) below the VCL, indicating a much more limited emittance growth during beam formation from the thicker photocathode. The results presented here highlight a key difference between the single-electron approach and the high throughput implementation in designing UEM. Because of the highly density-dependent phase space evolution, a delicate adaptation in optical control must be identified for each beam setting in order to properly focus the beam in space and time for implementing high-intensity UEM systems.

**TABLE II.** Longitudinal phase space parameters found using the atomic grating approach.

Photocathode coating (Ag on sapphire) (nm)	Photoemission mode	Number of electrons per bunch *N_e_* (×10^6^)	Normalized emittance^42^ *ε_*z*_* (*μ*m)	Pre-RF phase space chirp *a_z_* (ps^−1^)	Pre-RF bunch-width, RMS *σ_0_* (ps)
10	TPPE	17	1.5 ± 0.5	0.00010	18.2
10	TPPE	1.7	0.4 ± 0.2	0.00020	17.1
50	SPPE	1.0	0.03 ± 0.02	0.00041	5.8
30	SPPE	1.0	≤0.02	0.00038	4.9
30	SPPE	10	≤0.2	0.00040	12.2

**FIG. 7.**
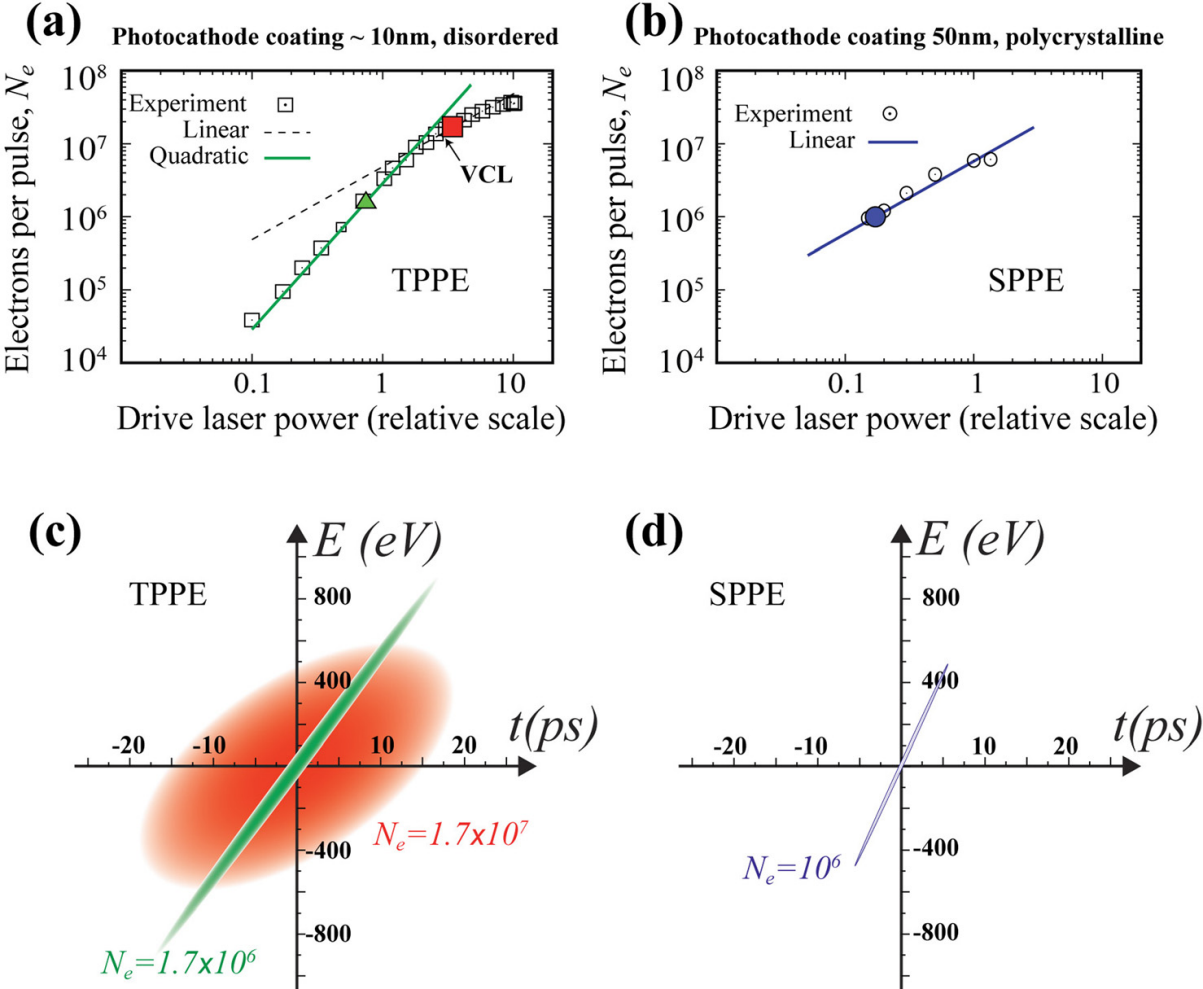
Characterization of photoemission scenarios and the phase space structures obtained from prototype case studies. (a) The emitted bunch electron numbers *N_e_* versus the drive laser power using an ultrathin silver photocathode coating (∼10 nm) on the sapphire window that results in a disordered film in this case. From fitting, the trend of emission before the VCL follows the quadratic dependence associated with the two-photon photoemission (TPPE). The solid symbols mark the cases where the RF compression experiments are reported in Fig. 6. (b) *N_e_* versus the drive laser power using a thicker silver coating (50 nm). The linear trend of the dependence indicates the scenario of single photon photoemission (SPPE). The solid symbol marks the case of SPPE presented in Fig. 6. (c) The pre-RF phase space structures for the cases of TPPE highlighted in panel (a). Their phase space parameters (listed in Table II) are extracted from the RF compression experiments. (d) The pre-RF phase space structure for the selected case of SPPE.

To understand the origin of these marked differences from the two photocathodes, we examined the mechanism of photoemission by inspecting its dependence on the drive laser power. The results, as depicted in Fig. 7, indicate very different pictures for the two cases. For the ultrathin photocathode as presented in Fig. 7(a), the emitted electron number follows a quadratic dependence until the VCL (*N_c_* ∼ 1.5 × 10^7^), which is established when the slope of electron yield starts to change. This quadratic dependence shows that the electrons generated from the ultrathin photocathode are via two-photon photoemission (TPPE). In contrast, for the 50 nm photocathode as presented in Fig. 7(b), the dependence is linear, indicating that the emission is through single-photon emission (SPPE). Returning to the cases reported earlier in the corresponding emission cases, marked by the solid symbols in Figs. 7(a) and 7(b), we construct their pre-RF phase space structures in Figs. 7(c) and 7(d), based on their refined parameters (Table II). We can immediately see a major difference in the phase space area determined for TPPE and SPPE cases. In the case of TPPE, the phase space associated with the above-VCL case [red Gaussian envelope in Fig. 7(c)] shows a significant stochastic nature where the correlation between energy and time for the emitted electrons is almost lost compared to the below-VCL case [green envelope in Fig. 7(c)]. This phenomenon may be associated with the generation of turbulent flow as described in the earlier MLFMM simulation^42^ due to initial charge pinning on the surface, acerbated by the early onset of the virtual cathode effect. This may be rationalized from the fact that the metal film does not perfectly wet the sapphire surface in our ultrathin film preparation, therefore photoemission may emerge from substrate-metal interfaces, including those metal-insulator surface states.^54^ Under this circumstance, the restricted subsurface counter charge movements produce a highly non-uniform field distribution near the surface that leads to a turbulent flow and an increase in emittance. This is in contrast to the laminar flow regime where the counter charges are free to expand along with the emitted electrons.^42^ Indeed, using the 50 nm photocathode coating [blue envelope in Fig. 7(d)], the phase space structure is highly correlated in comparison, resulting in generally higher beam brightness for a similar number of electrons being emitted.

We further investigated the performance by implementing the time compression measurements on the same TaS_2_ film^21^ after the energy compression experiments. The data from the time compression experiments are presented in Fig. 6(b). In particular, we find the difference between *η_E_* (indicated at the upward triangles) and *η_t_* (downward triangles) presented on the scale of $P_{RF}^{1/2}$ is quite similar for all cases [within 10%, see Fig. 6(a)]. Furthermore, we find that the analytical predictions (solid lines) from the same set of input parameters (Table II) used to fit the energy compression data presented in Fig. 6(a) can indeed consistently describe the datasets from time compression experiments [Fig. 6(b)]. While we do not completely rule out the interaction effect during compression, this near constant difference between *η_t_* and *η_E_* suggests that the linear model does a reasonable job in describing the RF compression optics for different density regimes presented here.

## VII. HIGH-BRIGHTNESS MODE AND THE CASE STUDY OF PHASE TRANSITION OF NANOSCALE VO_2_ CRYSTALS

The studies of photoinduced phase transition in VO_2_ nanocrystals represent a particularly challenging case for UED for various reasons. First, in order to avoid the buildup of transient stress during rapid structural transformation, the preparation of isolated samples on the nanometer scale is preferred.^55^ This puts a constraint on the electron dose and the repetition rate. For example, on the granular nanoscale VO_2_ films, the weak signals from key UED reflections relevant to the structural phase transition stand on a large diffusive scattering background. Furthermore, a low repetition rate is frequently required for the thermal relaxation of the isolated samples (>1 ms).^56^ All of these demand a high instantaneous dose, and to clearly track individual reflections from complex structural changes, a large coherence length is also typically required. On the other hand, the phase transition of VO_2_ is known to be potentially very fast especially in the ultrafine and unconstrained samples driven by the above-threshold photoexcitation. Such a high-speed transition serves well to test the performance limits of temporal compression at high beam intensities.

**FIG. 8.**
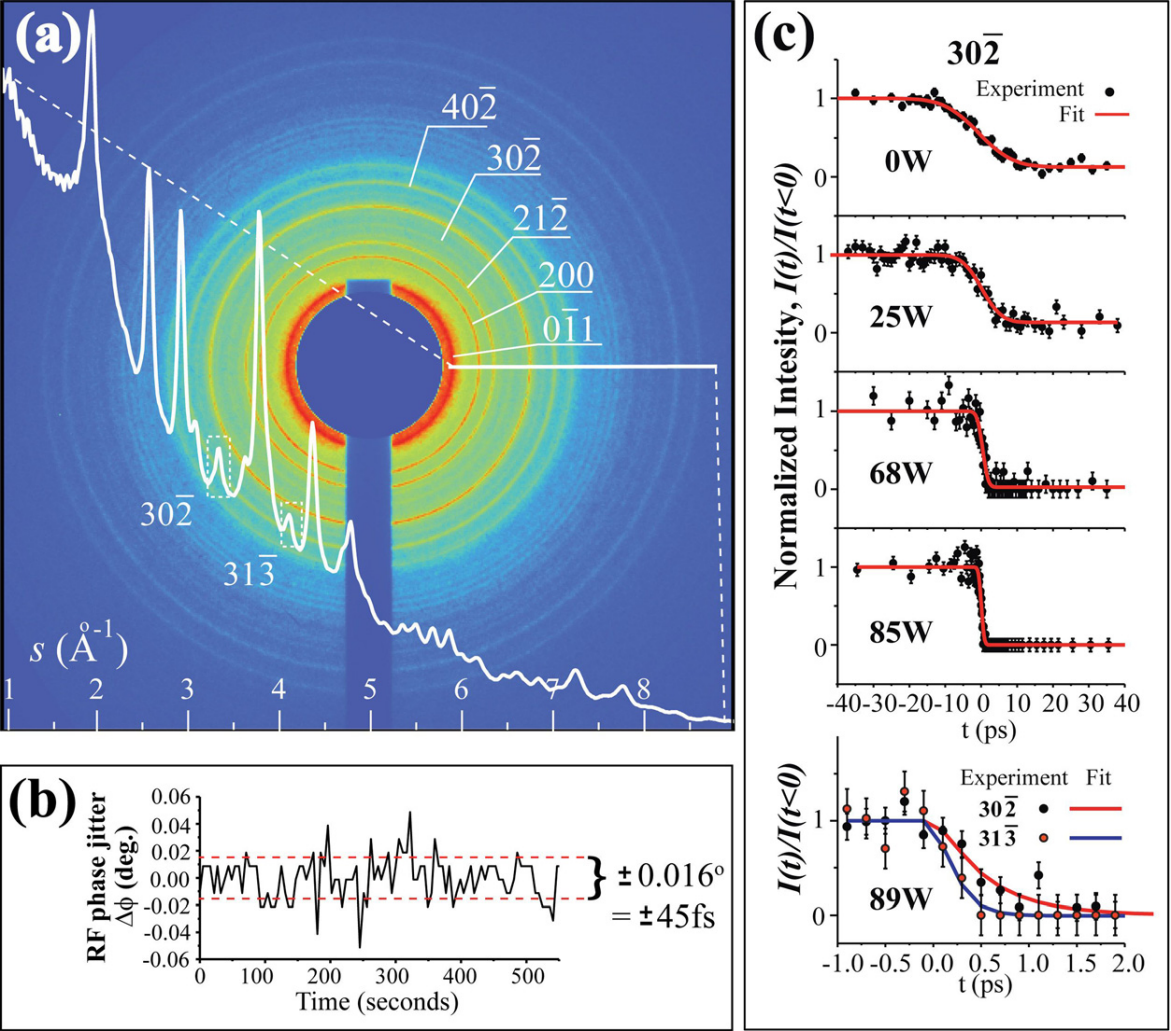
Ultrafast electron diffraction (UED) experiments on VO_2_ films conducted using the high-brightness electron beams. (a) The diffraction image obtained with *N_e_* = 10^7^. The superimposed diffraction curve is obtained through radially averaging the diffraction intensity from the range specified by the white horizontal bar. The indices for the key reflections are presented. (b) The RF phase jitter Δϕ recorded during the UED experiments. (c) The trace of $30\bar{2}$ reflection normalized intensity observed in the UED experiments with *N_e_* = 10^6^. The trace is fitted with an error function, where the resolution-limited transition time is used to determine the bunch duration at different RF powers. The lowest panel shows the case near optimal compression where the bunch duration is shorter than the response time of the experiments, allowing the different lattice dynamics projected along [$30\bar{2}$] and [$31\bar{3}$] directions to be resolved.

For this study, we implemented a 30 nm silver photocathode coating, matching the penetration depth of the drive laser. We drive the photoemission to achieve *N_e_* = 10^6^ and 10^7^ for case studies to elucidate the performance versus the dose. By adjusting the transverse lens pair (TL2 and TL3), we seek to tune the electron dose (*D_e_*) and the spatial coherence length (*L_C_*), where, for a given brightness, $L_{C}\propto D_{e}^{1/2}$.^57^ We set the TL2 and TL3 first for the *N_e_* = 10^7^ case, with a goal of delivering a coherent beam while maintaining a high dose. This is accomplished by creating a crossover very close to TL3 and using TL3 to produce a nearly parallel beam at the specimen by minimizing the lateral width at diffraction peaks. To better match the beam waist with the specimen size, a 150 *μ*m aperture [Aperture in Fig. 1(a)] is further employed, resulting in a beam waist of ∼65 *μ*m at the specimen. Figure 8(a) shows the diffraction image obtained with over 4000 shots from a 40 nm VO_2_ film, which is deposited on a 100 *μ*m × 100 *μ*m TEM nano-membrane window.^56^ From electron counting, we determine *D_e_* = 6.8 e/*μ*m^2^. Meanwhile, the coherence length is ≥15 nm, which is determined based on *L_C_ =λ_e_*/(2*α_s_*), where *α_s_*, the half width at half maximum of the beam divergence angle at the specimen, is estimated to be no more than 0.007°. We note that *α_s_* is retrieved based on *σ_B_* recorded on the camera (at *s *=* *7 Å^−1^) without excluding the finite inherent diffraction width from the VO_2_ film (which is appreciable due to inhomogeneity^56^) and energy-spread-led broadening, representing an upper limit of beam divergence. Correspondingly, we may estimate the transverse emittance for the incident beam contributing to the observed diffraction pattern. At the parallel beam waist, the normalized transverse emittance $\varepsilon_{x,y}∼\left(\gamma \sigma_{x,y}v_{e}\alpha_{B}\right)/\left(\sqrt{2\ln2}c\right)$ = 0.0034 *μ*m, where *σ_x,y_* is taken as 50 *μ*m according to the specimen size. This combined dose and coherence length corresponds to a 4D brightness *B_4D_*, defined by *N_e_*/(*ε_x_ε_y_*),^58^ in excess of 5 × 10^9^ *μ*m^−2^.

To evaluate the signal-to-noise ratio (*S/N*) requirement for the UED experiments, we consider the Poisson noise associated with the key weak reflections, such as $30\bar{2}$ and $31\bar{3}$, whose intensities are used as the order parameters to gauge the transitions.^52,54^ Specifically, the integrated intensity of these peaks is at the level of 10^−4^ of the overall number of electrons entering the specimen, *N_e_*′ =*D_e_*×(sample area). Moreover, the noise associated with the specific reflection intensity comes from the total intensity at the corresponding wavevectors (3.3 and 4.1 Å^−1^, respectively), including the major contribution from the background diffusive scattering (*N_Bkg_*) that is about 30 times larger than the signal retrieved from within the diffraction peak envelope (*N_hkl_*)—see Fig. 8(a). Therefore, *S/N* of the experiments $N_{hkl}/\sqrt{N_{Bkg}+N_{hkl}}$ is calculated to be ∼2 × 10^−3^*N_e_*′. Further complication comes from the low repetition rate (*f_Rep_* = 100 Hz) for ensuring the full thermal recovery during the pump-probe cycle in such a system.^56^ Therefore, for the 4000 shots, taken for 40 s of integration at *N_e_* = 10^7^, where *N_e_*′ exceeds 2.5 × 10^8^, the effective *S/N* is about 20. Reducing *N_e_* to 10^6^, which is required to reach sub-100 fs resolution according to our projections [Fig. 3(f)], the dose suffers by a factor of 4 under the same transverse optic settings. Correspondingly, the effective *S/N* is reduced to 10. Such fluctuations (∼10%) are indeed generally observed in the time-dependent measurements conducted with a similar level of *N_e_*′ as presented in Fig. 8(c). These transient responses recorded here, while not superb compared to typical UED experiments integrated over much longer pump-probe cycles, are nonetheless adequate to characterizing the bunch phase space structure.

In time-resolved experiments, VO_2_ films were optically pumped with 50 fs, 800 nm laser pulses at a fluence of ∼8 mJ/cm^2^. This selected fluence is about 15% above the previously identified threshold to promptly drive the phase transition from the monoclinic to the rutile state.^56^ Specifically, the inherent timescale for the phase transition ($\delta t_{i}$) is within 100 fs, set by the timescale of band gap collapse under an intense laser pulse.^59–61^ For a bunch duration that is much longer than 100 fs, it thus can be reasonably determined via fitting the order parameter responses using an error function, as shown in the upper panels of Fig. 8(c). However, near the time compression point, the compressed electron bunch may reach a timescale lower than $\delta t_{i}$. This is observed in the lower panel of Fig. 8(c), where different decay times of $30\bar{2}$ and $31\bar{3}$ are shown, resolving the inherently distinct dynamics previously suggested at ultrashort timescales in forming a metastable phase of VO_2_ [see Fig. 4(b) of Ref. 56].

**FIG. 9.**
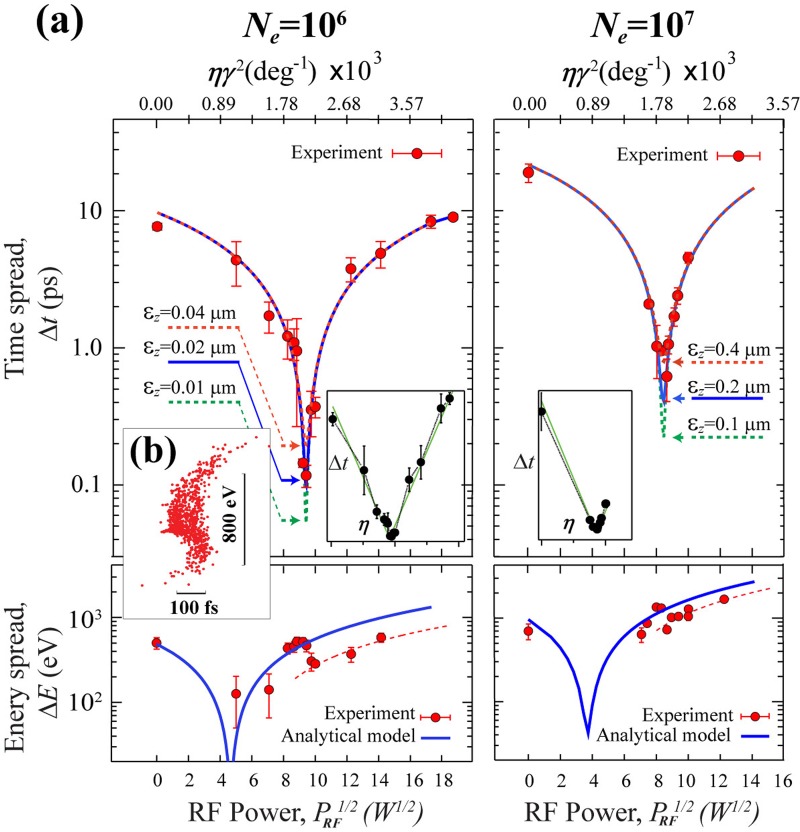
Phase space evolution of high-brightness beams under RF optics. (a) The RMS time (*Δt*) and RMS energy (*ΔE*) spreads of the electron bunches at *N_e_* = 10^6^ and 10^7^. The experimental data are depicted in solid symbols, whereas the lines are simulation results from the analytical model. The solid line represents the best fit to the experimental data. The logarithmic scale in the vertical axis highlights the values near the compression point, which is the most sensitive range for determining the emittance *ε_z_*. The insets show the data plotted in linear scales along with the best fits obtained using the analytical model (green). A visible deviation from the analytical model prediction is identified in the energy compression experiments presented in the lower part of the panels where the data after the time compression points collectively shift downward. This deviation can be attributed to the transformation of the phase space structure induced by the space charge effects at the temporal focal plane. (b) The phase space structure at the temporal focal plane obtained for *N_e_* = 10^6^ using the MLFMM approach on an exaggerated scale along the time-axis is shown. An S-shape distortion that leads to a contraction of energy spread is visible.

In terms of testing the performance limits of the bunches, we conducted the energy and time compression experiments with *N_e_* = 10^6^ and 10^7^. The results are presented in Fig. 9(a), where progression to reach sub-ps time compression is evident in both cases. Using the analytical model [see lines in Fig. 9(a) and the insets showing the linear scale], we fit the data to obtain the phase space parameters. Especially, the bunch durations obtained near the compression points are used to evaluate the emittance. For *N_e_* = 10^6^, the shortest RMS bunch duration accomplished is 120 fs at near 90 W. We note that the RF timing jitter, $\tau_{RF}=\Delta \varphi_{RMS}/f_{0}$∼45 fs, is determined based on the RMS phase jitter Δφ RMS ∼ 0.016° [Fig. 8(b)]. However, more pertinently, the resolution in the pump-probe experiments is determined by the arrival time fluctuations of the electron bunches, determined by $\tau_{Ar}=\eta \Delta \varphi_{RMS}L/v_{e}$ (≤ 100 fs for compression power ≤100 W). From the apparent minimum RMS bunch duration of 120 fs, we determine *ε_z_* = 0.02 ± 0.01 *μ*m for the *N_e_* = 10^6^ case. This is expected to be an upper limit, as after deconvoluting the inherent timescale of the phase transition (*δt_i_* ∼ 80 fs),^59,60^ the actual RMS bunch duration could be less than 100 fs.^30^ For *N_e_* = 10^7^, the shortest bunch obtained at near 75 W is around 500 fs, which, when compared with the simulations, corresponds to *ε_z_* ∼ 0.2 *μ*m. Remarkably, these experimentally characterized emittances at the specimen, which is 1.4 m away from the cathode, are merely a factor of 2 larger than the emittances calculated near the source using the MLFMM method.^42^ These results indicate a high-level of emittance preservation along the beam column. For comparison, the experimental results from different regimes and their associated phase space parameters are summarized in Table II.

We further examine the nonlinearities that may occur during bunch compression. While the linear structure of the electron bunch phase space enables high-efficiency compression of the bunch by nearly two orders of magnitude [Fig. 9(a)], subtle phase space structural changes are also observed after the temporal crossovers. These are more clearly shown in the corresponding energy spread data, depicted in the lower part of Fig. 9(a), where a downward shift of *ΔE* is observed after reaching the respective time compression point. This phenomenon is also seen in Fig. 6(a) for beams generated under different scenarios. However, the cases are more distinct for the high-brightness beams where a tighter crossover is accomplished. An explanation for this behavior may be drawn from the MLFMM results for the *N_e_* = 10^6^ case [see Fig. 3(f)], which is depicted in an enlarged view in Fig. 9(b). On this enhanced scale, the correlation between the energy and the time coordinates of the bunch at the crossover is no longer linear, but rather has an S-shape. This phase space structure can be modeled by including the 3rd-order and 5th-order aberration coefficients. We note that these nonlinearities could not be produced by the curvature effect from the RF optics even by exaggerating its impact—the nonlinear RF field would lead to an opposite bending of the phase space structure. In fact, we expect a very small nonlinearity from the RF optics because the bunch duration observed in our studies is generally much smaller than the RF period (∼1 ns). Instead, such nonlinearities may be driven by the collective space charge forces under a tight focusing. At the time compression point, the electrons at two opposite ends of the bunches may encounter resistive forces by the electrons at the core, whereas the electrons close to the core may receive a push, hence contributing to the velocity profile along the bunch. Examining the projected *σ_z_* and *σ_vz_* from the curved phase space structure, indeed, suggests that such nonlinear effects can cause *ΔE* to decrease and *Δt* to increase as compared to the linear scenarios that qualitatively explain the observed trends in our experiments. We note that these nonlinearities do not occur significantly prior to the time compression, and therefore do not severely impede the optimization of the ultrafast diffraction or spectroscopy experiments using the compression optics.

## VIII. CONCLUSIONS

We have demonstrated flexible uses of RF longitudinal optics to energetically and temporally condense the high-intensity electron beams at the specimen for a range of beam densities near the virtual cathode limit. Specifically, the ability to reduce the energy spread to the emittance-limit of a few eV through phase space manipulation offers the opportunity to produce nearly monochromatic beams while preserving the beam intensity. This mechanism is very different from the monochromator currently implemented in TEMs that reduces the energy spread mainly by slicing out the electrons beyond the resolution window, leading to reduced beam intensity. This new capability is central for implementing ultrafast electron energy loss spectroscopy, where a synergistic combination of two RF lenses can be used for simultaneously achieving high temporal and spectral resolutions.^38^ It may be interesting to point out that, in the most favorable cases presented here, the deliverable 6D brightness, defined by *N_e_*/*(ε_x_ε_y_ε_z_*),^42,58^ is close to 10^12^ e/*μ*m^3^. This is equivalent to a bunch brilliance approaching 10^20^ electrons/(mrad^2^mm^2^·s·eV), which is comparable to the most advanced synchrotron sources^62^ in effective scattering strength, when considering the scattering cross-section of electrons being 4–5 orders of magnitude higher than that of X-rays. While this may be expected in the steady state as has been demonstrated utilizing the field emission source in TEMs,^2^ such a high performance has not been proven experimentally for the space-charge-dominated beams at a sub-relativistic energy. This preservation of linear phase space structures and their emittance opens a pathway for designing high performance femtosecond electron microscopes utilizing high-brightness beams at typical TEM energy scales. To this end, the methodology of atomic grating characterization and the effective modeling outlined here may provide useful feedback for the optical design and the performance, which may be essential, given the diverse phase space parameters observed.

The VO_2_ experiments as demonstrated here highlight the need for a high-throughput electron beamline that could simultaneously provide the required high instantaneous dose, coherence length, and temporal resolution, which may be offered by employing a high-brightness beam, to resolve the sensitive physics that are beyond the reach of the beamline relying on a higher repetition rate. The current performance in the high-brightness modes identified here is subject to the specific design of our photogun. We expect that beams with higher brightness may be generated by improving the initial conditions for streamlining the photoemission, including using pulse shaping to form more homogeneous wavefronts and time structures of the drive laser pulses, and increasing the extraction field at the cathode. Nonetheless, following the favorable scaling of emittance over a reduced emitted electron density, we expect that even higher temporal and energy resolutions can already be accomplished here simply by deploying fewer electrons to reduce the beam emittance. Indeed, from our comparative studies, we show that the longitudinal emittance scales nearly linearly with respect to *N_e_*, which is supported by the N-particle bunch compression simulations [Fig. 3(f)] and the previous source emittance studies.^42^ We therefore expect much shorter bunches, at the 10 fs level, or a lower energy spread, at 1 eV or less, to be delivered to the specimen using 10^5^ or fewer electrons. This scaling between the source emittance and *N_e_* is consistent with the recent measurements conducted by the RF streaking experiments for RF-compressed beams with *N_e_* ∼ 10^5^ (Ref. 9) and *N_e_* ∼ 10^6^.^30^ In the lower density regimes (*N_e_* <10^6^), however, the practical resolution in the UED experiments is expected to be limited by the short-time phase jitters in our PLL design, which is currently at ∼45 fs (RMS) [Fig. 8(b)]. For general deployment, the state-of-the-art PLL design has reached less than 1 fs timing precision between the laser and RF systems,^45,63^ which can also be implemented here. However, a more pertinent issue is the longer time jitter incipient to the low frequency instabilities in the environment. This is crucial when a longer acquisition time is necessary for the most demanding experiments. While our temperature stabilization scheme using a second cavity PLL drastically helps the long-term stability in a less ideal laboratory setting, it cannot correct the cumulative room temperature drift that may ultimately exceed the stability window. To this end, such long-time-scale shift may be corrected by using the recorded phase as a time stamp to reconstruct the arrival times of the bunches for each individual image frame based on the *in situ* determined RF focusing strength (*η*). Furthermore, the non-interacting analytical model will need to be modified with an additional repulsive term as an effective theory for modeling the performance of compression optics in the more intensely focusing regimes, for example, with a shorter focusing distance, for microdiffraction or imaging experiments. These aspects will be discussed separately elsewhere.

## References

[1] R. Henderson , Q. Rev. Biophys. 28, 171 (1995). 10.1017/S003358350000305X 7568675

[2] P. W. Hawkes and J. C. H. Spence , *Science of Microscopy* ( Springer, New York, 2007), Vol. I.

[3] R. Srinivasan , V. A. Lobastov , C. Y. Ruan , and A. H. Zewail , Helv. Chim. Acta 86, 1761 (2003). 10.1002/hlca.200390147

[4] B. J. Siwick , J. R. Dwyer , R. E. Jordan , and R. J. D. Miller , J. Appl. Phys. 92, 1643 (2002). 10.1063/1.1487437

[5] B. W. Reed , J. Appl. Phys. 100, 034916 (2006). 10.1063/1.2227710

[6] S. P. Weathersby , G. Brown , M. Centurion , T. F. Chase , R. Coffee , J. Corbett , J. P. Eichner , J. C. Frisch , A. R. Fry , M. Gühr , N. Hartmann , C. Hast , R. Hettel , R. K. Jobe , E. N. Jongewaard , J. R. Lewandowski , R. K. Li , A. M. Lindenberg , I. Makasyuk , J. E. May , D. McCormick , M. N. Nguyen , A. H. Reid , X. Shen , K. Sokolowski-Tinten , T. Vecchione , S. L. Vetter , J. Wu , J. Yang , H. A. Dürr , and X. J. Wang , Rev. Sci. Instrum. 86, 073702 (2015). 10.1063/1.4926994 26233391

[7] Y. Murooka , N. Naruse , S. Sakakihara , M. Ishimaru , J. Yang , and K. Tanimura , Appl. Phys. Lett. 98, 251903 (2011). 10.1063/1.3602314

[8] J. Li , W.-G. Yin , L. Wu , P. Zhu , T. Konstantinova , J. Tao , J. Yang , S.-W. Cheong , F. Carbone , J. A. Misewich , J. P. Hill , X. Wang , R. J. Cava , and Y. Zhu , Npj Quantum Mater. 1, 16026 (2016). 10.1038/npjquantmats.2016.26

[9] J. Maxson , D. Cesar , G. Calmasini , A. Ody , P. Musumeci , and D. Alesini , Phys. Rev. Lett. 118, 154802 (2017). 10.1103/PhysRevLett.118.154802 28452517

[10] G. Sciaini and R. J. D. Miller , Rep. Prog. Phys. 74, 096101 (2011). 10.1088/0034-4885/74/9/096101

[11] C. Gerbig , A. Senftleben , S. Morgenstern , C. Sarpe , and T. Baumert , New J. Phys. 17, 043050 (2015). 10.1088/1367-2630/17/4/043050

[12] C.-Y. Ruan , Y. Murooka , R. K. Raman , R. A. Murdick , R. Worhatch , and A. Pell , Microsc. Microanal. 15, 323 (2009). 10.1017/S1431927609090709 19575833

[13] R. C. Dudek and P. M. Weber , J. Phys. Chem. A 105, 4167 (2001). 10.1021/jp010122t

[14] A. H. Zewail , Annu. Rev. Phys. Chem. 57, 65 (2006). 10.1146/annurev.physchem.57.032905.104748 16599805

[15] A. Hanisch-Blicharski , A. Janzen , B. Krenzer , S. Wall , F. Klasing , A. Kalus , T. Frigge , M. Kammler , and M. Horn-von Hoegen , Ultramicroscopy 127, 2 (2013). 10.1016/j.ultramic.2012.07.017 22975358

[16] C.-Y. Ruan , V. A. Lobastov , F. Vigliotti , S. Y. Chen , and A. H. Zewail , Science 304, 80 (2004). 10.1126/science.1094818 15064414

[17] C.-Y. Ruan , Y. Murooka , R. K. Raman , and R. A. Murdick , Nano Lett. 7, 1290 (2007). 10.1021/nl070269h 17397235

[18] B. J. Siwick , J. R. Dwyer , R. E. Jordan , and R. J. D. Miller , Science 302, 1382 (2003). 10.1126/science.1090052 14631036

[19] J. Cao , Z. Hao , H. Park , C. Tao , D. Kau , and L. Blaszczyk , Appl. Phys. Lett. 83, 1044 (2003). 10.1063/1.1593831

[20] M. Eichberger , H. Schaefer , M. Krumova , M. Beyer , J. Demsar , H. Berger , G. Moriena , G. Sciaini , and R. J. D. Miller , Nature 468, 799 (2010). 10.1038/nature09539 21107321

[21] T.-R. T. Han , F. Zhou , C. D. Malliakas , P. M. Duxbury , S. D. Mahanti , M. G. Kanatzidis , and C.-Y. Ruan , Sci. Adv. 1, e1400173 (2015). 10.1126/sciadv.1400173 26601190 PMC4640616

[22] K. Haupt , M. Eichberger , N. Erasmus , A. Rohwer , J. Demsar , K. Rossnagel , and H. Schwoerer , Phys. Rev. Lett. 116, 016402 (2016). 10.1103/PhysRevLett.116.016402 26799033

[23] A. H. Zewail , Science 328, 187 (2010). 10.1126/science.1166135 20378810

[24] *Advances in Imaging and Electron Physics*, edited by Anatoli A. I. and Sergei A. A. ( Elsevier, 2014), Vol. 184.

[25] V. A. Lobastov , R. Srinivasan , and A. H. Zewail , Proc. Nat. Acad. Sci. U. S. A. 102, 7069 (2005). 10.1073/pnas.0502607102 PMC1129142 15883380

[26] Z. Su , J. S. Baskin , W. Zhou , J. M. Thomas , and A. H. Zewail , J. Am. Chem. Soc. 139, 4916 (2017). 10.1021/jacs.7b00906 28273420

[27] B. Barwick , D. J. Flannigan , and A. H. Zewail , Nature 462, 902 (2009). 10.1038/nature08662 20016598

[28] A. Feist , N. Bach , N. Rubiano da Silva , T. Danz , M. Möller , K. E. Priebe , T. Domröse , J. G. Gatzmann , S. Rost , J. Schauss , S. Strauch , R. Bormann , M. Sivis , S. Schäfer , and C. Ropers , Ultramicroscopy 176, 63 (2017). 10.1016/j.ultramic.2016.12.005 28139341

[29] T. T. A. Lummen , R. J. Lamb , G. Berruto , T. LaGrange , L. Dal Negro , F. J. G. de Abajo , D. McGrouther , B. Barwick , and F. Carbone , Nat. Commun. 7, 13156 (2016). 10.1038/ncomms13156 27725670 PMC5062594

[30] T. van Oudheusden , P. L. E. M. Pasmans , S. B. van der Geer , M. J. de Loos , M. J. van der Wiel , and O. J. Luiten , Phys. Rev. Lett. 105, 264801 (2010). 10.1103/PhysRevLett.105.264801 21231672

[31] R. P. Chatelain , V. R. Morrison , C. Godbout , and B. J. Siwick , Appl. Phys. Lett. 101, 081901 (2012). 10.1063/1.4747155

[32] M. Gao , H. Jean-Ruel , R. R. Cooney , J. Stampe , M. de Jong , M. Harb , G. Sciaini , G. Moriena , and R. J. D. Miller , Opt. Express 20, 12048 (2012). 10.1364/OE.20.012048 22714191

[33] J. S. Kim , T. LaGrange , B. W. Reed , M. L. Taheri , M. R. Armstrong , W. E. King , N. D. Browning , and G. H. Campbell , Science 321, 1472 (2008). 10.1126/science.1161517 18787163

[34] L. Stojchevska , I. Vaskivskyi , T. Mertelj , P. Kusar , D. Svetin , S. Brazovskii , and D. Mihailovic , Science 344, 177 (2014). 10.1126/science.1241591 24723607

[35] R. M. van der Veen , T. J. Penfold , and A. H. Zewail , Struct. Dyn. 2, 024302 (2015). 10.1063/1.4916897 26798793 PMC4711615

[36] R. F. Egerton , *Electron Energy-Loss Spectroscopy in the Electron Microscope* ( Plenum, New York, 1996).

[37] S. G. Anderson , P. Musumeci , J. B. Rosenzweig , W. J. Brown , R. J. England , M. Ferrario , J. S. Jacob , M. C. Thompson , G. Travish , A. M. Tremaine , and R. Yoder , Phys. Rev. Spec. Top. - Accel. Beams 8, 014401 (2005). 10.1103/PhysRevSTAB.8.014401

[38] F. Zhou , J. Williams , and C.-Y. Ruan , Chem. Phys. Lett. 683, 488 (2017). 10.1016/j.cplett.2017.03.019

[39] H. H. Rose , *Geometrical Charged-Particle Optics* ( Springer-Verlag London Ltd, Godalming, 2009), Vol. 142, p. 1.

[40] K. Chang , Ph.D. thesis, Michigan State University, 2014.

[41] A. Valfells , D. W. Feldman , M. Virgo , P. G. O'Shea , and Y. Y. Lau , Phys. Plasmas 9, 2377 (2002). 10.1063/1.1463065

[42] J. Portman , H. Zhang , Z. Tao , K. Makino , M. Berz , P. M. Duxbury , and C.-Y. Ruan , Appl. Phys. Lett. 103, 253115 (2013). 10.1063/1.4855435

[43] J. H. Billen and L. M. Young , Los Alamos National Laboratory Report No. LA-UR-96-1834, posson superfish, 1996.

[44] T. Wangler , *RF Linear Accelerators* ( Wiley-VCH Verlag GmbH & Co. KGaA, Weinheim, 2004).

[45] Y. Jun , H. Schnatz , and L. W. Hollberg , IEEE J. Sel. Top. Quantum Electron. 9, 1041 (2003). 10.1109/JSTQE.2003.819109

[46] K. Floettmann , Phys. Rev. Spec. Top.-Accel. Beams 6, 034202 (2003). 10.1103/PhysRevSTAB.6.034202

[47] P. Zhang , A. Valfells , L. K. Ang , J. W. Luginsland , and Y. Y. Lau , Appl. Phys. Rev. 4, 011304 (2017). 10.1063/1.4978231

[48] Z. Tao , H. Zhang , P. M. Duxbury , M. Berz , and C.-Y. Ruan , J. Appl. Phys. 111, 044316 (2012). 10.1063/1.3685747

[49] H. Zhang and M. Berz , Nucl. Instrum. Methods Phys. Res., Sect. A 645, 338 (2011). 10.1016/j.nima.2011.01.053

[50] H. Zhang , Z. Tao , C.-Y. Ruan , and M. Berz , Adv. Imaging Electron Phys. 191, 56 (2015). 10.1016/bs.aiep.2015.03.012

[51] A. M. Michalik and J. E. Sipe , J. Appl. Phys. 99, 054908 (2006). 10.1063/1.2178855

[52] J. Portman , H. Zhang , K. Makino , C.-Y. Ruan , M. Berz , and P. M. Duxbury , Adv. Imaging Electron Phys. 191, 117 (2015).

[53] K. Makino and M. Berz , Nucl. Instrum. Methods Phys. Res., Sect. A 558, 346 (2006). 10.1016/j.nima.2005.11.109

[54] T. Lewowski , Thin Solid Films 259, 53 (1995). 10.1016/0040-6090(94)06392-3

[55] Z. Tao , T.-R. T. Han , S. D. Mahanti , P. M. Duxbury , F. Yuan , C.-Y. Ruan , K. Wang , and J. Q. Wu , Phys. Rev. Lett. 109, 166406 (2012). 10.1103/PhysRevLett.109.166406 23215102

[56] Z. Tao , F. Zhou , T.-R. T. Han , D. Torres , T. Wang , N. Sepulveda , K. Chang , M. Young , R. R. Lunt , and C.-Y. Ruan , Sci. Rep. 6, 38514 (2016). 10.1038/srep38514 27982066 PMC5159834

[57] C.-Y. Ruan , P. M. Duxbury , and M. Berz , “ Ultrafast nonlinear imaging and spectroscopy II,” Proc. SPIE 9198, 91980Q (2014).

[58] J. Portman , H. Zhang , K. Makino , C.-Y. Ruan , M. Berz , and P. M. Duxbury , J. Appl. Phys. 116, 174302 (2014). 10.1063/1.4900582

[59] B. T. O'Callahan , A. C. Jones , J. Hyung Park , D. H. Cobden , J. M. Atkin , and M. B. Raschke , Nat. Commun. 6, 6849 (2015). 10.1038/ncomms7849 25897640

[60] D. Wegkamp , M. Herzog , L. Xian , M. Gatti , P. Cudazzo , C. L. McGahan , R. E. Marvel , R. F. Haglund , A. Rubio , M. Wolf , and J. Stähler , Phys. Rev. Lett. 113, 216401 (2014). 10.1103/PhysRevLett.113.216401 25479507

[61] A. Cavalleri , T. Dekorsy , H. H. W. Chong , J. C. Kieffer , and R. W. Schoenlein , Phys. Rev. B 70, 161102 (2004). 10.1103/PhysRevB.70.161102

[62] J. Ullrich , A. Rudenko , and R. Moshammer , Ann. Rev. Phys. Chem. 63, 635 (2012). 10.1146/annurev-physchem-032511-143720 22404584

[63] A. Kalaydzhyan , M. Y. Peng , M. Xin , K. Shafak , W. Wang , and F. X. Kärtner , J. Phys.: Conf. Ser. 741, 012084 (2016). 10.1088/1742-6596/741/1/012084

